# Two Surgeons' Technique for Laparoscopic Cholecystectomy in Situs Inversus for a Right-Handed Surgeon: Technical and Ergonomic Considerations

**DOI:** 10.7759/cureus.38161

**Published:** 2023-04-26

**Authors:** Venu Bhargava M, Venkata Vineeth Vaddavalli, Kishore Abuji, Pranay Palle, Krishna Ramavath

**Affiliations:** 1 Department of Surgical Gastroenterology, Employees' State Insurance Corporation (ESIC) Medical College and Hospital, Hyderabad, IND; 2 Department of General Surgery, Postgraduate Institute of Medical Education and Research, Chandigarh, IND; 3 Department of General Surgery, Gandhi Medical College and Hospital, Hyderabad, IND; 4 Department of General Surgery, All India Institute of Medical Sciences, Hyderabad, IND

**Keywords:** cholecystectomy, gall bladder, cholecystitis, dextrocardia, situs inverts

## Abstract

Laparoscopic cholecystectomy can be technically challenging in patients with situs inversus totalis. A middle-aged gentleman presented with pain in the left upper abdomen. His cardiac workup showed dextrocardia, and ultrasonography showed a gall bladder on the left side. He was diagnosed with acute cholecystitis and was planned for laparoscopic cholecystectomy. We used the four-port technique, where anterior dissection was carried out by the dominant right hand of the primary surgeon, and the infundibulum was retracted by the first assistant from the mid-clavicular port. The first assistant carried out the posterior dissection through a midclavicular port, whereas the primary surgeon did a retraction. To conclude, this technique done by two surgeons decreases the ergonomic difficulty faced by right-handed surgeons while performing laparoscopic cholecystectomy.

## Introduction

Situs inversus totalis is a rare congenital anomaly with an estimated incidence of 1 per 5000-20,000 live births [1]. Anatomically, the gall bladder is located in the left hypochondrium, posing a diagnostic challenge to suspect symptomatic gallstone disease clinically. Laparoscopic surgery in these patients poses technical challenges, especially for right-handed surgeons, like dissecting the Calot's triangle with the non-dominated hand and the chances of crossing the arms [1]. Another concerning issue in these patients is associated with vascular anomalies, which are common in patients with situs inversus [2,3]. Despite these concerns, laparoscopic cholecystectomy is the treatment of choice and can be performed safely even in acute cholecystitis patients with situs inversus [4]. In this case presentation, we discussed the technical and ergonomic considerations for safe cholecystectomy in patients with situs inversus.

## Case presentation

**Figure 1:**
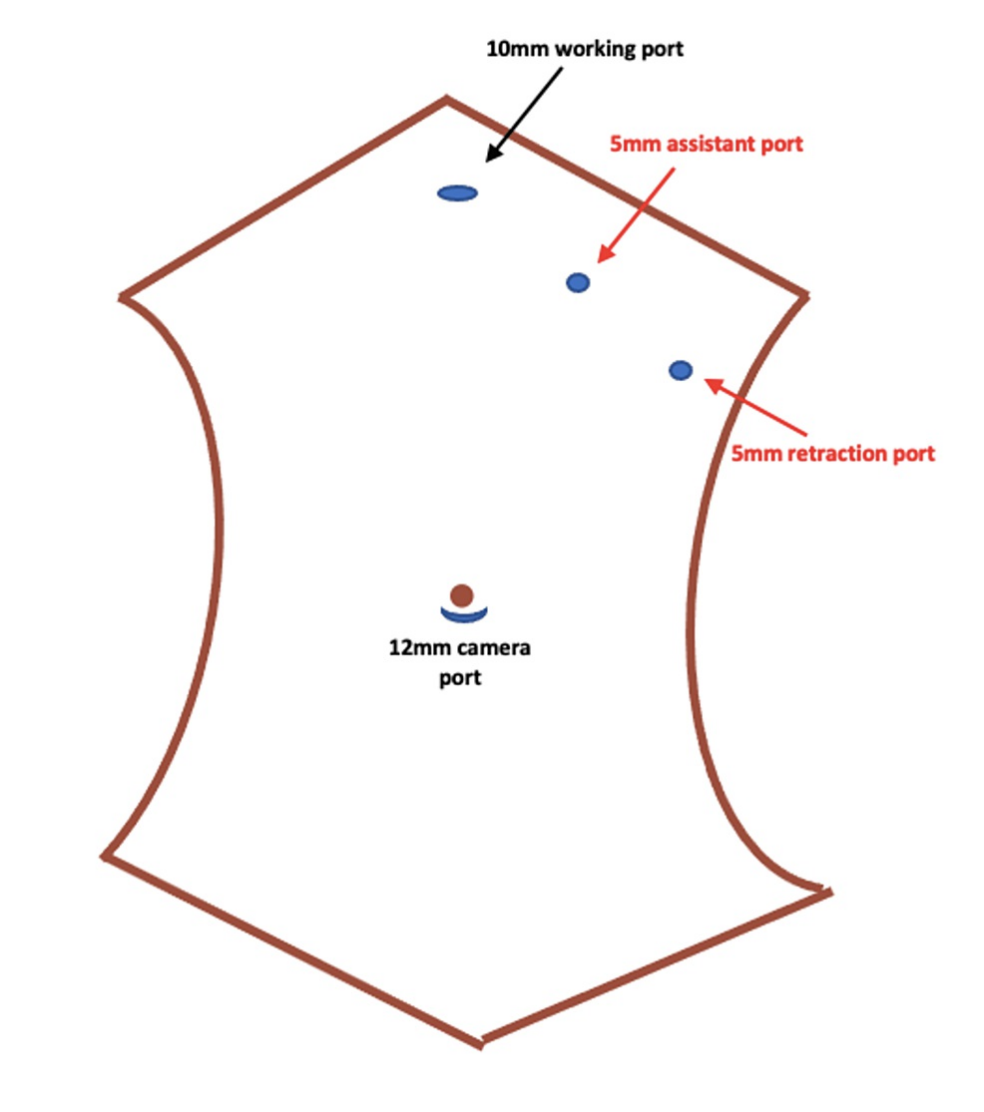
Image depicting the port placement in situs inversus

A middle-aged gentleman complained of pain in the left hypochondrium for one month. Ultrasonography revealed a spleen in the right hypochondriac region and a gallbladder in the left hypochondriac region, and stones were identified in the gallbladder. Based on ultrasonography findings, a diagnosis of symptomatic gallstones was made. His laboratory parameters, electrocardiogram (ECG), and echocardiography were normal, and he was planned for laparoscopic cholecystectomy. We started the procedure by adjusting the theatre equipment on the left side, mirroring the right side. The primary surgeon and first assistant stood on the patient's right side and the second assistant on the left side. Four ports were used. Two 10 mm ports were placed in the subxiphoid region towards the left and the other supra umbilically. Two 5 mm ports were placed in the left hypochondriac region, one in the left midclavicular line and the other in the left anterior axillary line at the level of the umbilicus (Figure 1). 

An open technique was used to create a pneumoperitoneum by the surgeon standing on the patient's left side. The abdomen was inspected, and a gall bladder was found on the left side.

**Figure 2:**
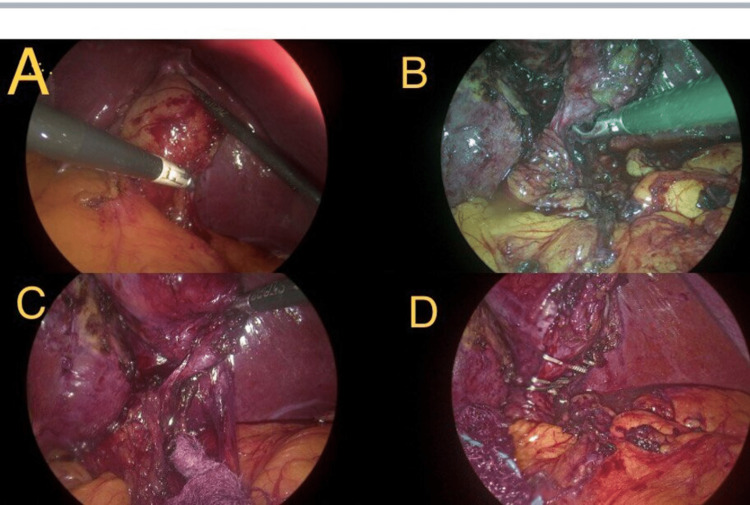
Intraoperative images showing the technique of dissection A: Right hand of the primary surgeon using Maryland for separating adhesions while retraction by the assistant. B: Posterior dissection is being done by the right hand of the assistant surgeon using Maryland dissecting forceps. C: Critical view of safety achieved. D: Clips were placed.

To prevent cross-over of the arms of the operating surgeon, the assistant on the left retracted the fundus of the gallbladder. By using the Maryland dissecting forceps, Calot's triangle was dissected, the anterior fibrofatty layer was dissected by the primary surgeon (Figure 2A), and posterior dissection was carried out by the first assistant to prevent cross-over of the arms (Figure 2B). Once Calot's triangle was dissected and a critical view of safety was achieved (Figure 2C), the cystic artery and the cystic duct were clipped and divided. The gall bladder was dissected from the liver surface using hook diathermy by the first assistant, and traction was given by the primary surgeon. The gall bladder was delivered from the epigastric port, closed ports using non-absorbable sutures. 

The total operating time was 60 minutes, and the postoperatively patient was discharged the next day following surgery. Postoperatively no complications were noted to date. 

## Discussion

The right-handed surgeon might experience technical difficulties while performing the laparoscopic cholecystectomy in situs inversus totalis patients. The difficulty might be due to a) difficult adhesiolysis with the non-dominant hand, b) constant mental orientation of the anatomy, and c) cross-over of the hands while dissecting the Calot's triangle [5-7]. Dissection of the Calot's triangle would be easier for a left-handed surgeon than a right-handed surgeon [8-10].

Many techniques are described to decrease the difficulty, like the four-port technique, the French position of the operating surgeon, frequent changes in instruments between two arms, retracting infundibulum through the sub-xiphoid port, and port relocation [11-13]. These techniques, even though helpful, could not prevent cross-over of the arms leading to an abnormal posture for the surgeon.

The two surgeons' technique can be followed to avoid difficult dissection, where the primary and the first assistant use the dominant right hand [12,14]. However, the problem with this technique is that it requires good coordination among the operating surgeons to prevent abnormal posture for the operating surgeon [15]. In this technique, the primary surgeon dissects the Calot's triangle with their dominant hand, while the infundibulum is retracted by the first assistant and the fundus by the second assistant [16-18]. The first assistant carries out posterior dissection while the operating surgeon retracts the infundibulum.

Dissection by the left hand from the sub-xiphoid port makes the instrument lose its perpendicular axis and creates a narrow angle, making dissection difficult {18]. However, this problem can be overcome by doing anterior dissection alone from the dominant right hand of the right-handed surgeon and posterior dissection by the first assistant through the mid-clavicular port. This technique's advantages include using the surgeon's dominant hand, preventing abnormal postures when the surgeon is standing to the patient's left, and simplifying the procedure ergonomically. Bile duct injuries following laparoscopic cholecystectomy have been rarely reported and can be prevented using similar techniques described when the gall bladder is on the right side [19,20].

## Conclusions

To conclude, laparoscopic cholecystectomy can be performed safely, even in the presence of situs inversus. Some technical modifications, like two surgeons' technique, might decrease the ergonomic and technical difficulties faced by the right-handed surgeon in the presence of situs inversus. However, to standardize these techniques, a prospective study might be required, although because of the rarity of situs inversus, this might not be possible.
